# Parallel Structure Deep Neural Network Using CNN and RNN with an Attention Mechanism for Breast Cancer Histology Image Classification

**DOI:** 10.3390/cancers11121901

**Published:** 2019-11-29

**Authors:** Hongdou Yao, Xuejie Zhang, Xiaobing Zhou, Shengyan Liu

**Affiliations:** School of Information Science and Engineering, Yunnan University, Kunming 650091, China

**Keywords:** breast cancer, biopsy image, DenseNet, LSTM, attention, switchable normalization, targeted dropout, test time augmentation

## Abstract

In this paper, we present a new deep learning model to classify hematoxylin–eosin-stained breast biopsy images into four classes (normal tissues, benign lesions, in situ carcinomas, and invasive carcinomas). Our model uses a parallel structure consist of a convolutional neural network (CNN) and a recurrent neural network (RNN) for image feature extraction, which is greatly different from the common existed serial method of extracting image features by CNN and then inputting them into RNN. Then, we introduce a special perceptron attention mechanism, which is derived from the natural language processing (NLP) field, to unify the features extracted by the two different neural network structures of the model. In the convolution layer, general batch normalization is replaced by the new switchable normalization method. And the latest regularization technology, targeted dropout, is used to substitute for the general dropout in the last three fully connected layers of the model. In the testing phase, we use the model fusion method and test time augmentation technology on three different datasets of hematoxylin–eosin-stained breast biopsy images. The results demonstrate that our model significantly outperforms state-of-the-art methods.

## 1. Introduction

**Figure 1:**
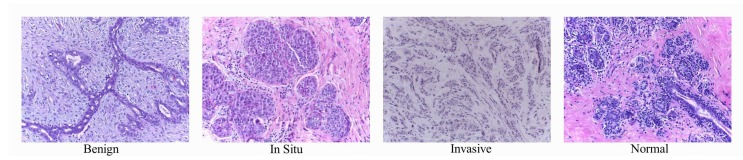
Examples of microscopic biopsy images.

Currently, the incidence of breast cancer is increasingly high. Breast cancer has become the most common type of cancer that threatens human health, especially in women, whose incidence of breast cancer is much higher than that of men. Many researchers have started to devote themselves to developing a high-performance and reliable computer-aided system to help medical staff diagnose breast cancer using histopathological microscopic images and improve diagnostic efficiency. The diagnostic process of breast cancer is not only time-consuming and expensive but also largely dependent on the consistency of pathologists’ existing knowledge and pathological reports. The development of computer vision and deep learning provides a more reliable classification method for the histological evaluation of hematoxylin–eosin-stained sections. These methods can classify breast tissue with high accuracy. Therefore, researchers have developed many image analysis algorithms for breast cancer detection tasks [1,2,3,4]. However, according to the current research status of the algorithm, the performance of these algorithms is far from meeting clinical requirements. Therefore, researchers continue to develop new algorithms for histopathological image analysis. It is hoped that these algorithms can accurately classify an image as normal tissue, non-malignant (benign) lesions, in situ carcinomas, or invasive breast cancer. Figure 1 shows some examples of histopathological images of breast cancer. It contains four categories of human breast cells: normal tissues, non-malignant tumors, in situ carcinomas, and invasive carcinomas.

In the field of deep learning computer vision, convolutional neural networks (CNNs) are commonly used, whereas few people use recurrent neural networks (RNNs). In the early stages of this field, some people tried to combine CNNs and RNNs to solve the general image classification problem. The most straightforward method is to use a CNN to extract histology image features and then input the extracted features into an RNN [5,6,7]. The method proposed by Zheng et al. [8] and the method proposed by Nahid et al. [9] flatten the feature of the last layer of the convolutional neural network to one dimension and then input the result into the recurrent neural network. Hua’s [10] method is similar to Zheng’s. Zhao et al. introduced channel-wise attention [11]. Koo et al. [12] used the features extracted from the intermediate convolution layer as input to the recurrent neural network. In a three-dimensional image processing study, Shi et al. [13] also performed feature extraction by a 3D convolutional neural network and used the extracted features as input to a hierarchical recurrent neural network. The design concept of combining CNN and RNN models was first applied to the general image domain, and it was then extended to the field of histology image processing. There are similar methods in the field of medical image processing [9,14,15,16]. Long Short-Term Memory(LSTM) can learn not only the short-term memory between features but also the long-term memory between features. Researchers began to design a parallel structure of CNN and RNN for image processing. Jiang Wang et al. [17] used a CNN and RNN in a parallel structure design model for the multi-label image classification task. Yin et al. [18] used a CNN and RNN to extract the features of the image, but they used simple concatenating operations to process the features extracted from the two different networks. This is a simple and effective method that ensures the desired effect and reduces the calculation. Gaobo Liang et al. [15] also used the CNN and RNN structure to process images in parallel. To process the features from the two different networks, they used element-wise multiplication for feature merging.

The most well-known and influential human breast cancer datasets include BACH2018 [19], Bioimaging2015 [20], and extended Bioimaging2015 [21]. Meng et al. [22] proposed a gravitational loss (G-loss) as a new loss function to maximize interclass differences and minimize intraclass variance. Their approach uses Resnet with ImageNet weight as the basis of the network and the G-Loss function to optimize the model. Their model achieved the best results on the BACH2018 dataset. On the Bioimaging2015 dataset, Vo’s method [23] achieved a 96.4% accuracy for the four classifications. Alom et al. [24] designed an inception recurrent residual convolutional neural network (IRRCNN) model and achieved a 98.59% testing accuracy for multiclass breast cancer recognition. In addition, they achieved 100% testing performance in an experiment in which the classification model was applied to random patches, followed by a winner-take-all method to produce the final results. To date, this is the best result on the Bioimaging2015 dataset. Yan [21] fine-tuned the Inception V3 network as an image feature extractor and inputted the extracted features into an LSTM model. Yan’s method achieved the best (82.1%) patch-wise accuracy on the extended Bioimaging2015 dataset.

Motivated by the previous studies, we designed a parallel structure of a CNN and RNN for the classification of hematoxylin–eosin-stained breast biopsy images. We obtained state-of-the-art results on three human breast cancer datasets.

## 2. The Proposed Model

In the proposed model, the RNN is considered to have the same ability as the CNN to extract features from images, i.e., the CNN and RNN have the same important position. After obtaining two different kinds of features by the CNN and RNN, respectively, the model uses the perceptron attention mechanism to weigh the features so that the model can better trade off two kinds of features extracted by the CNN and RNN in the training process.

Finally, targeted dropout [25] replaces the traditional dropout method in the fully connected layers. It uses a pruning strategy so that the model can more purposeful suppresses different neurons.

**Figure 2:**
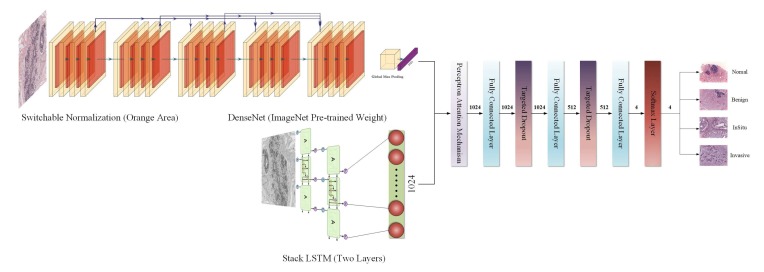
The overall structure of the model designed in our study.

The overall structure of our proposed model is shown in Figure 2.

### 2.1. The CNN Module

The CNN has a powerful feature extraction ability. As the depth of the CNN structure increases, the problem of gradient disappearance becomes more and more obvious. Dense connections operation of the DenseNet [26] is one of the excellent solutions to this issue. However, dense connections are likely to cause an increase in the channel dimension. In order to solve the problem of too many parameters, the author uses a 1∗1 convolution structure in front of each dense block layer to reduce the dimension, so that no matter how many channels are input, the output of each layer is the same value. Then, the network parameters of DenseNet are greatly reduced. Further, this dense connection is equivalent to that each layer directly connects input and loss, which alleviates the problem of gradient disappearance when calculating the loss function. In this paper, we used DenseNet as the CNN module to extract RGB three-channel medical image features.

### 2.2. Switchable Normalization

The switchable normalization method, which is the most recent normalization method. It does not design a new normalization algorithm but unifies the existing instance normalization, layer normalization, and batch normalization.

Assuming that the format of feature map in the model is (N,C,H,W), where *N* represents the batch size, *C* represents the number of channels, *H* represents the height of feature map, *W* represents the width of the feature map. Each pixel is represented by $h_{ncij}$ and the subscript ncij represents the index in the direction of each dimension. Then each pixel is normalized by switchable normalization, and the normalized pixel value ${\tilde{h}}_{ncij}$ is obtained.

(1) $${\tilde{h}}_{ncij}=\gamma \frac{h_{ncij}-\sum_{k\in \Omega }\omega_{k}\mu_{k}}{\sqrt{\sum_{k\in \Omega }\omega_{k}^{\prime }\sigma_{k}^{2}+\epsilon }}+\beta$$

where γ denotes scaling coefficients, while β denotes deviation coefficient, which is similar to the definitions of other normalization methods. The biggest difference is the mean μ and variance $\sigma^{2}$ in switchable normalization. They are not calculated in layers or channels, but on a set of Ω, the set of Ω is BN,LN,IN three normalization methods. The method of the weighted average is equivalent to using one attention mechanism in three normalization methods.

Switchable normalization method can enable a model to learn the most suitable normalization for each layer in the process of training [27]. We used this new switchable normalization method instead of the classical batch normalization method as the normalization method in the CNN module.

### 2.3. The RNN Module

It is generally acknowledged that an image has spatial characteristics. From the perspective of the pixel level, there is a time sequence relationship between the pixels of the image. If the width of the image is regarded as the eigenvalue and the height of the image as the time step, then each row of pixels in each image can be considered to have a temporal relationship. Therefore, when designing the model, we considered not only the spatial relationship of the image but also the time sequence relationship between the pixels. We used stacked LSTM [28] as the RNN module to extract time sequence features of the pixels.

### 2.4. The Attention Mechanism

Because we use the CNN and RNN modules in a parallel structure to design the model, we can obtain the different kinds of features extracted by the two modules. But how can we let the model to process these features and decide which kind of features is most important contribution to classification? For this purpose, we sought help from the methods in the field of natural language processing and the perceptron attention mechanism [29] was added after obtained features extracted by the CNN and RNN modules. Then, the model can learn the appropriate weight between the two features by itself and dynamically allocate it in the training process.

(2) $$\begin{array}{c} a=\;\sigma \left(tanh\left(W_{cnn}*f_{cnn}+W_{rnn}*f_{rnn}\right)\right)\\ F=\;a*f_{cnn}+\left(1-a\right)*f_{rnn} \end{array}$$

where $f_{cnn}$ denotes the features of the RGB medical image obtained by the CNN module, $f_{rnn}$ denotes the features of the gray medical image obtained by the RNN module, and $W_{cnn}$ and $W_{rnn}$ denote the weight matrix of corresponding features of the convolutional neural network and the recurrent neural network, respectively. *F* is the final eigenvalue of the model and is calculated by the attention mechanism. With the learned weight, the model can focus its attention on the eigenvalue of the appropriate module and dynamically allocate the proportion of the features of the CNN and RNN modules. As a result, the model has a better performance and can better adapt to small datasets. Thus, it is more robust.

### 2.5. Targeted Dropout

In the proposed approach, the latest targeted dropout method is used between the fully connected layers in the last part of the model. Each time the back-propagation algorithm updates the network weight, the targeted dropout method employs strategies to filter a group of candidate weights to be updated, and then it applies this set of candidate weights to the general dropout for the random pruning operation. Finally, the model can learn how to prune during the training process to improve the robustness of the model and further enhance model performance.

## 3. Datasets

We tested our model on three different human breast cancer datasets to establish its performance. 

**BACH2018 dataset** [19]

The 15th International Conference on Image Analysis and Recognition provided a very large annotated dataset consisting of two parts: one was the breast cancer histology (BACH2018) dataset, and the other was whole-slide images (WSI). The microscopic images in the BACH2018 dataset were hematoxylin–eosin-stained histopathological mammary microscopic images. Four categories were labeled in the dataset: Normal, Benign, Insitu, and Invasive. The whole dataset was composed of 400 training images and 100 test images; the training set contained 100 images in each category for a uniform distribution. All images were captured in 2014, 2015, and 2017 using a Leica DM 2000 LED microscope and a Leica ICC50 high-definition camera. All patients were from Covilh and Porto (Portugal). Two medical experts annotated the images, and normal and benign inconsistent images were discarded. The remaining suspicious cases were determined by immunohistochemical analysis. The image format is RGB.tiff. The size of the image is 2048 × 36 pixels, with a pixel scale of 0.42 × 0.42 μm. The corresponding training set label of the image is given in the form of a .CSV file.

**Bioimaging2015 dataset** [20]

Under the same sampling conditions, the Bioimaging 2015 breast histology classification challenge dataset contained digitized hematoxylin–eosin-stained histological images with an image size of 2048 × 1536 pixels and an in-plane pixel size of 0.42 × 0.42 μm. Each image was marked by two pathologists, who provided diagnoses according to the content of the image and divided each image into four categories: normal tissue, benign lesions, carcinoma in situ, and invasive cancer. A total of 249 microscopic training images and 36 microscopic test images were recorded.

**Extended Bioimaging2015 dataset** [21]

This dataset was an extension of the Bioimaging2015 dataset and contained 1319 hematoxylin and eosin color images that were high resolution (2048 × 1536 pixels), uncompressed, and annotated. All images were digital, and the acquisition conditions were the same: the magnification is 200×, and the pixel size is 0.42 × 0.42 μm. The standard preparation procedure was used in this project. The paraffin method is widely used in clinics. Each image was marked as normal, benign, in situ, or invasive cancer according to the main type of cancer in the image. The annotations were completed by two medical experts, and divergent images were discarded.

### 3.1. Data Preprocessing

We carried out separate experiments on the BACH2018 dataset, Bioimaging2015 dataset, and extended Bioimaging2015 dataset. The BACH2018 dataset has 100 histological images in each category in the training set and a total of 100 histological images in the test set. The Bioimaging2015 dataset has 249 histological images in the training set and 36 histological images in the test set. The extended Bioimaging2015 dataset has 1319 histological images, and there is no test set.

Medical images require the use of specific image normalization methods to remove the influence of stains on the biological tissues. Marc Macenko et al. [30] proposed a standardized method for the quantitative analysis of tissue slices, and it takes into account the staining techniques used to prepare the tissue slices. First, logarithmic transformation is used to transform the color of the image into an optical density. Then, singular value decomposition is applied to the OD tuple to obtain a 2D projection with a large variance. Then, generated color space transformation is applied to the original image. Finally, the histogram of the image is stretched to dynamically cover more than 90% of the data.

We performed color normalization on the BACH2018 and Bioimaging2015 datasets, except the extended Bioimaging2015 dataset. Because the latter has a large number of images and good sampling quality.

### 3.2. Data Extension

For a deep learning model, datasets are crucial. The number and distribution of a dataset and the difference in each category affect the performance of the model. The numbers of the three datasets are very small, and each histological image is a 2048×1536 high-resolution image. It is not suitable to train complex neural networks with the original-sized images because it requires extensive computing, and computer hardware cannot bear such high computing costs. Therefore, we further processed these three datasets.

**Random Cropping:** Because the numbers of three datasets are all small, we expanded the dataset by random cropping of a 2048×1536 normalized image to 50 1024×768 patches.

**Scaling:** Since the original image is a 2048×1536 high-resolution image, we cannot directly use the original size of the image to train the neural network model because it will cause memory overflow. Even the 1024×768 image size after random cropping is still too large for the training model. Therefore, we further used a linear interpolation algorithm to scale the cropped images. We zoomed 1024×768 images to 256×192 images without changing the ration of the original tissue structure.

### 3.3. Data Augmentation

The aim of data augmentation is to reduce the influence of latent irrelevant factors on the model as much as possible for training a deep neural network. This requires we should further process the preprocessed datasets.

**Stain augmentation:** Stain augmentation is a unique augmentation method for medical images. The image data augmentation method proposed by Arnout C. Ruifrok et al. was used in this work. The purpose of this method is to establish a histochemical staining separation method of the immune system by using color image analysis technology.

**Rotation:** Rotation is another way of data augmentation which the picture is randomly rotated at a certain angle during the training process. We used the rotation method and set the rotation angle range between 0 and 360 degrees to improve the generalization performance of the model.

**Translation:** Translation is another data augmentation method. It randomly translates images to a certain distance during training.

**Flip:** In image flipping, there is a certain probability that an image is flipped in the training process. We used both horizontal and vertical flips.

**Grayscale:** The purpose of graying is to transform the three-channel color images into single-channel gray-white images, thereby eliminating the image channels and reducing the dimensionality. In this study, RGB color medical images were processed by averaging the values of the three R, G, B channels to a gray value because the RNN module can only receive two-dimensional data without channels.

## 4. Testing Technology

We tested the performance of our model on the BACH2018, Bioimaging2015, and extended Bioimaging2015 datasets. Since BACH2018 and Bioimaging2015 have no validation sets, we used the *k*-fold method on these two datasets and used 20% of the training set as a validation set. It was divided into five folds altogether. For the convenience of comparison, we randomly selected 400 pictures from 1319 pictures and used 70% as the training set, 20% as the validation set, and 10% as the test set. The *k*-fold method was not used in training.

In the test phase, we performed the same way as the official method which used the single model prediction and model integration methods for the prediction, and we also used the test time augmentation (TTA) method to augment the test data. The soft voting and hard voting methods were used to predict the test data. Here, we briefly introduce the methods used in our experiments.

### 4.1. Model Ensemble

We used the *k*-fold method on the BACH2018 and Bioimaging2015 datasets. We divided the datasets into five folds, trained 50 epochs for each fold, and saved the model after each epoch. According to the accuracy of the model on the validation set during the training phase, the best-performing model on the validation set was selected from each fold. There were five best models selected for integrated prediction.

### 4.2. TTA

In order to reduce the influence of the diversity of test data, TTA technology was introduced in the prediction phase. It is a simple data enhancement of the test set in the test stage included rotation, clipping, scaling, and other operations; thus, a single original image results in images with multiple enhancements. This process makes the model more comprehensively consider the potential possibilities of a picture in the test, so it makes its final judgment by synthesizing various factors.

### 4.3. Soft Voting

We used TTA technology to crop 100 areas randomly for each test image. Each region was forecasted by the trained model, and 100 groups of predicted values were finally obtained. The probability values of these 100 groups were then averaged. The label with the largest predicted value was the final predicted category of the model.

### 4.4. Hard Voting

Each trained model arrived at the predicted value of a test picture by soft voting. A total of five models received five groups of predicted labels. These five models then determined the final predicted label of the test picture by the principle of the minority being subordinate to the majority.

## 5. Parameter Settings and Experimental Results

The purpose of our experiment was to prove that the model structure we designed had great advantages, rather than relying on fine-tuning to improve experimental results, and fine-tuning is time-consuming, so we did not perform fine-tuning in this paper. All experiments described in this paper used the same default initialization parameters: the batch size was 20, the momentum was 0.9, the initial learning rate was 0.02, and the optimizer was Adam.

To evaluate our model’s performance, we used accuracy, precision, recall, and F1-Score indicators on Bioimaging2015 and extended Bioimaging2015 datasets. The BACH2018 dataset didn’t release golden labels of the test set, and could only be evaluated online, so we only used the accuracy indicator provided by official to evaluate our model.

### 5.1. BACH2018 Dataset

Using the whole source size of image to train the model is unrealistic. It not only causes high computing cost, but also brings burden to the computer hardware. First of all, we shrank the source image and determined what size of the image was the most effective to train the model. We shrank the picture into three different sizes, 256×192, 512×384 and 1024×768, to train an existing model and submitted the result to the official site.

As shown in Table 1, we selected Inception-ResNet V2 as the base model to test the effect of different size images on the training model. According to the results evaluated by official site, we found training model with the image size of 256×192 has the best performance.

Because there were only 400 images in the training set, we expanded the training set with the data extension method proposed above and the subsequent experiments were also based on the expanded data set.

We carried out the experiment step by step through the control variable method. The following table reports our experimental results on the BACH2018 dataset. In Table 2, SN—switchable normalization, and TD—targeted dropout.

**Figure 3:**
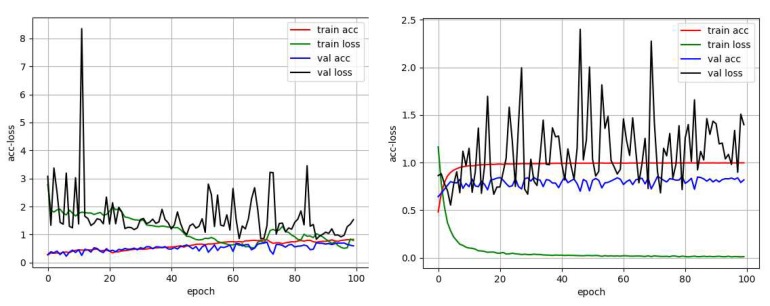
The left is the Acc-Loss schematic diagram of original DenseNet model, and the right is the Acc-Loss schematic diagram of the model designed in this study.

As shown in Table 2, the training accuracy of the original CNN (DenseNet) network on the dataset was not high; the highest accuracy of the *k*-fold was only about 0.7, and the accuracy on the test set (100 images) was 0.73. Then, the model gradually improved. With the original CNN (DenseNet) module as the basis, targeted dropout, switchable normalization, and LSTM were added one by one. The accuracy on the test set was higher than that of the original CNN (DenseNet) module. The accuracies on the test sets were more than 0.8; thus, the effect of the improved model was better than that of the original CNN (DenseNet) network structure. A comparison of the original DenseNet network performance with the training process performance of the improved complete model revealed that the improved network model was markedly better than the original network structure. The process of Acc-Loss is shown in Figure 3.

In Figure 3, the left side shows the original Acc-Loss curve of DenseNet trained by 100 epochs, and the right side shows the Acc-Loss curve of the improved model trained by 100 epochs. As observed in the two figures, the original DenseNet model converged very slowly and was not fully converged by 40 epochs. The model proposed in this paper tended to converge after training for 10 epochs. On the BACH2018 dataset, we chose the Inception ResNet V2 and Xception network as the CNN modules in this model for comparative experiments. We combined these three structures together and replaced one CNN module at a time to find the best convolution module. The results of numerous experiments showed that targeted dropout, switchable normalization, and LSTM were effective in the design model. Experiments also showed that the highest accuracy per fold of a complete network structure (only the CNN modules differed in each experiment) was more than 0.85. The DenseNet model used as a CNN module achieved an accuracy of 0.92 on the test set. This accuracy is 1% higher compared with the current best result of 0.91 [22].

### 5.2. Bioimaging2015 Dataset

The basic structure of the model was determined by experiments on the BACH2018 dataset. We further experimented on the Bioimaging2015 dataset and adjusted the RNN module in the model using different RNN structures, Gated Recurrent Unit(GRU) or LSTM. Through the experiment, we found that the best structure of the RNN module was LSTM. The experimental results are shown in Table 3.

**Figure 4:**
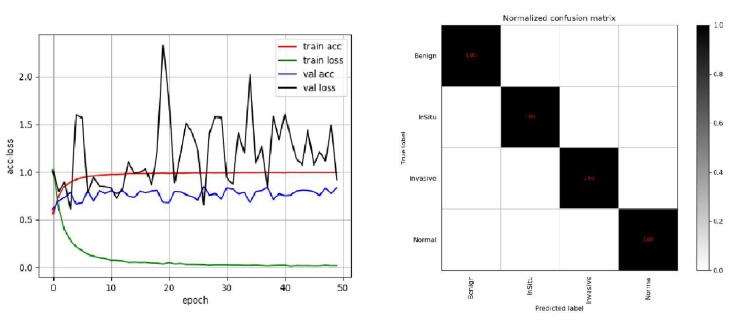
The schematic diagram of the Acc-Loss training process of our model and confusion matrix of the best result on Bioimaging2015 dataset.

**Figure 5:**
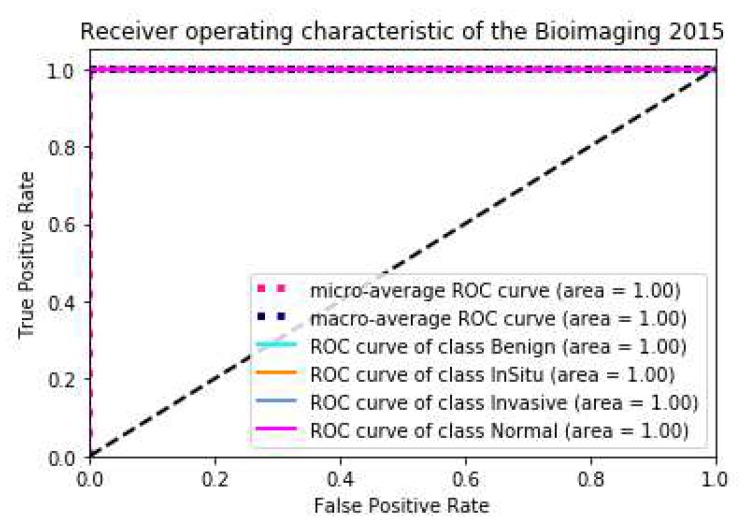
The ROC Curve of the result on Bioimaging2015 Test set (Best).

To compare conveniently with the best result published by Alom [24], we also used 5-fold models for integrated prediction on the test set and predicted five times in total. Among them, only one image was wrong for three predictions, and the accuracy rate was 97.2%; all images were correct for the other two prediction, and the accuracy rate was 1. The final average accuracy rate was 98.3%, which is higher than the results of Vo et al. presented in [23]. The best result was 1, which is the same as the best result of Alom et al. [24]. In order to reflect the performance of the model more directly, we draw the ACC loss curve of the training model, the confusion matrix and ROC curve on the test set, as shown in Figure 4 and Figure 5.

We also calculated the precision, recall and F1-Score on the Bioimaging2015 test set using the optimal model structure and tested it five times. The results are shown in Table 4, Table 5 and Table 6, respectively.

### 5.3. Extended Bioimaging2015 Dataset

**Figure 6:**
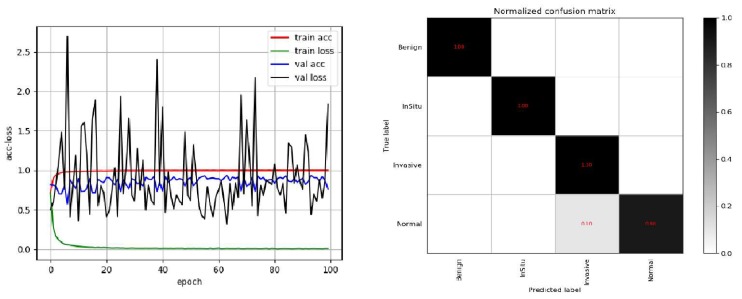
The schematic diagram of the Acc-Loss training process of the model and the confusion matrix of the best results on the extended Bioimaging2015 dataset [21].

**Figure 7:**
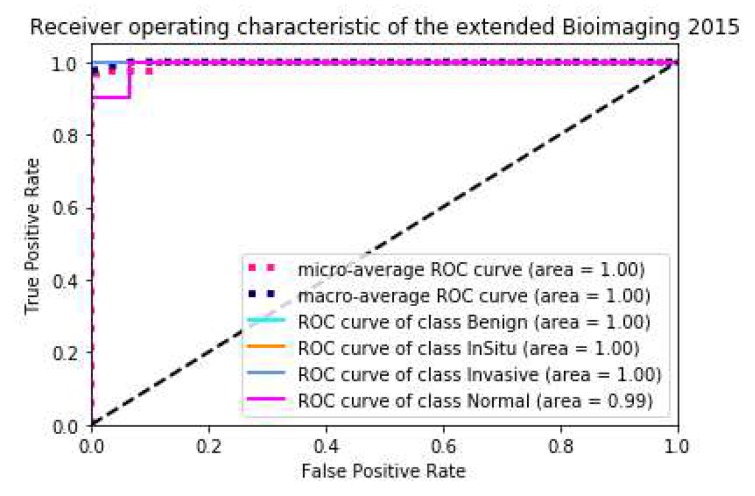
The ROC Curve of the result on extended Bioimaging2015 Test sets(single model) [21].

On the extended Bioimaging2015 dataset, instead of using the *k*-fold method, we trained the model directly on the pre-divided training set and then tested it on the pre-divided test set. We also used a single model and multi-modal integration to forecast directly. Table 7 showed that the state-of-the-art result on this dataset was achieved by only a single model. The prediction results of the five-model ensemble were consistent with those of the single model, with little difference. This result is mediocre. Because we did not use the *k*-fold method, the difference in the data and feature distribution between the trained models is small, and the effect of the ensemble is not obvious. The precision, recall and F1 score are shown in Table 8, Table 9 and Table 10, respectively, the ACC loss curve, confusion matrix and ROC curve are shown in Figure 6 and Figure 7, respectively.

## 6. Conclusions

In this paper, we propose a new deep learning model that uses two different types of neural networks—a CNN (DenseNet) and an RNN (LSTM)—with a special perceptron attention mechanism commonly used in NLP field [31] that unifies the image features extracted from the two different types of networks. The latest switchable normalization method and targeted dropout regularization technology are combined to improve the performance and robustness of the model. TTA method is introduced into our model during test phase. Our proposed model achieved state-of-the-art results on three datasets, which showed the advantage of parallel structure of deep neural network using CNN and RNN with attention mechanism. 

## Figures and Tables

**Table 1:** The prediction results from Inception-ResNet-V2 prediction for the different size of the BACH2018 dataset.

Accuracy	256×192	512×384	1024×768
CNN (Inception-ResNet V2)	0.83	0.77	0.71

**Table 2:** The prediction results from our model prediction for the four classifications of the BACH2018 dataset.

Models	1-Fold	2-Fold	3-Fold	4-Fold	5-Fold	Accuracy on Test Set (Best)
CNN (DenseNet121)	0.7312	0.6273	0.5973	0.7645	0.6795	0.73
CNN (DenseNet121) + TD	0.7593	0.7485	0.7062	0.7993	0.838	0.82
CNN (DenseNet121) + SN	0.868	0.8682	0.78	0.8635	0.8735	0.86
CNN (DenseNet121) + RNN (LSTM)	0.8397	0.7688	0.8128	0.8662	0.8482	0.82
CNN (DenseNet121) + SN + RNN (LSTM) + TD	0.8542	0.8495	0.8495	0.8355	0.811	**0.89**
CNN (DenseNet121) + SN + RNN (LSTM) + Attention + TD	0.8515	0.847	0.8148	0.8647	0.8323	**0.89**
CNN (Xception) + SN + RNN (LSTM) + Attention + TD	0.8113	0.8487	0.8143	0.863	0.8468	0.86
CNN (Inception ResNet V2) + SN + RNN (LSTM) + Attention + TD	0.8113	0.8487	0.8143	0.863	0.8468	0.86
CNN (DenseNet121 + Xception + Inception ResNet V2) + TD + SN + RNN (LSTM)+ Attention (Ensemble)						0.88
CNN (DenseNet121 + DenseNet169) + TD + SN + RNN (LSTM) + Attention (Ensemble)						**0.92**
ResNet + G-loss [22]						**0.91**

**Table 3:** The results of our model predicting four classes and the best published results on the Bioimaging2015 dataset.

Models	1-Fold	2-Fold	3-Fold	4-Fold	5-Fold	Accuracy (Best) on Test Set
CNN (DenseNet121) + SN + RNN (**GRU**) + Attention + TD	0.8067	0.8396	0.8148	0.8647	0.8323	0.86
CNN (DenseNet121) + SN + RNN (**LSTM**) + Attention + TD	0.8357	0.8659	0.8148	0.8647	0.8323	**1.00**
CNN (DenseNet121) + SN + RNN (**GRU+LSTM**) + Attention + TD	0.8157	0.8408	0.7959	0.7757	0.8498	0.94
CNN (Xception) + Gradient boosting trees (GBT)(Fusion) [23]						**0.96**
Inception Recurrent Residual Model [24]						**1.0**

**Table 4:** The precision score of our proposed model for each of the five predictions.

Model	Benign	In Situ	Invasive	Normal
CNN (DenseNet121) + SN + RNN (**LSTM**) + Attention + TD	1.0	1.0	1.0	1.0

**Table 5:** The recall score of our proposed model for each of the five predictions.

Model	Benign	In Situ	Invasive	Normal
CNN (DenseNet121) + SN + RNN (**LSTM**) + Attention + TD	1.0	1.0	1.0	1.0

**Table 6:** The F1-Score of our proposed model for each of the five predictions.

Model	Benign	In Situ	Invasive	Normal
CNN (DenseNet121) + SN + RNN (**LSTM**) + Attention + TD	1.0	1.0	1.0	1.0

**Table 7:** Our proposed methods and the best published results on a randomly partitioned test set.

Methods	Accuracy on the Test Set
Our Single Model	97.5%
Our Ensemble Model	97.5%
CNN + BiLSTM (serial architecture) [21]	82.1%

**Table 8:** The precision score for each of the five predictions (single model).

Model	Benign	In Situ	Invasive	Normal
CNN (DenseNet121) + SN + RNN (**LSTM**) + Attention + TD	1.0	1.0	0.909	1.0

**Table 9:** The recall score for each of the five predictions (single model).

Model	Benign	In Situ	Invasive	Normal
CNN (DenseNet121) + SN + RNN (**LSTM**) + Attention + TD	1.0	1.0	1.0	0.9

**Table 10:** The F1-Score for each of the five predictions (single model).

Model	Benign	In Situ	Invasive	Normal
CNN (DenseNet121) + SN + RNN (**LSTM**) + Attention + TD	1.0	1.0	0.952	0.947
